# Identification of Key Genes and Pathways in Triple-Negative Breast Cancer by Integrated Bioinformatics Analysis

**DOI:** 10.1155/2018/2760918

**Published:** 2018-08-02

**Authors:** Pengzhi Dong, Bing Yu, Lanlan Pan, Xiaoxuan Tian, Fangfang Liu

**Affiliations:** ^1^Tianjin State Key Laboratory of Modern Chinese Medicine, Tianjin University of Traditional Chinese Medicine, Tianjin 300193, China; ^2^Tianjin Central Hospital of Gynecology Obstetrics, Tianjin 300100, China; ^3^Department of Breast Pathology and Research Laboratory, Key Laboratory of Breast Cancer Prevention and Therapy (Ministry of Education), National Clinical Research Center for Cancer, Tianjin Medical University Cancer Institute and Hospital, Tianjin 300060, China

## Abstract

**Purpose:**

Triple-negative breast cancer refers to breast cancer that does not express estrogen receptor (ER), progesterone receptor (PR), or human epidermal growth factor receptor 2 (Her2). This study aimed to identify the key pathways and genes and find the potential initiation and progression mechanism of triple-negative breast cancer (TNBC).

**Methods:**

We downloaded the gene expression profiles of GSE76275 from Gene Expression Omnibus (GEO) datasets. This microarray Super-Series sets are composed of gene expression data from 265 samples which included 67 non-TNBC and 198 TNBC. Next, all the differentially expressed genes (DEGs) with p<0.01 and fold change ≥1.5 or ≤-1.5 were identified.

**Result:**

56 upregulated and 151 downregulated genes were listed and the gene ontology (GO) and Kyoto Encyclopedia of Genes and Genomes pathway (KEGG) enrichment analysis was performed. These significantly changed genes were mainly involved in the biological process termed prostate gland morphogenesis, inner ear morphogenesis, cell maturation, digestive tract morphogenesis, autonomic nervous system development, monovalent inorganic anion homeostasis, neural crest cell development, regulation of dendrite extension and glial cell proliferation, immune system process termed T cell differentiation, regulation of immune response, and macrophage activation. Genes are mainly involved in the KEGG pathway termed Oocyte meiosis. All DEGs underwent survival analysis using datasets from The Cancer Genome Atlas (TCGA) integrated by cBioPortal, of which amplification of SRY-related HMG-box 8 (SOX8), androgen receptor (AR), and Chromosome 9 Open Reading Frame 152 (C9orf152) were significantly negative while Nik Related Kinase (NRK) and RAS oncogene family 30 (RAB30) were positively correlated to the life expectancy (p<0.05).

**Conclusions:**

In conclusion, these pathways and genes identified could help understanding the mechanism of development of TNBC. Besides, SOX8, AR, C9orf152, NRK and RAB30, and other key genes and pathways might be promising targets for the TNBC treatment.

## 1. Introduction

Triple-negative breast cancer (TNBC) refers to the breast cancer that does not express the genes for estrogen receptor (ER), progesterone receptor (PR), or human epidermal growth factor receptor 2 (Her2/neu) [1]. It composed 15-20% of all breast cancers in the United States with poor prognosis [2]. Lack of expression of these receptors made it much more difficult to treat that it often requires a combination of therapies [3]. However, due to the absence of newly found targets, conventional chemotherapy was the main treatment used in clinical practice with suboptimal outcomes [4].

It is commonly accepted that TNBCs comprise heterogenous groups at the clinical [5, 6], histological [7, 8], and molecular levels [9–12]. Recently, genomic DNA copy number arrays, messenger RNA arrays, exon sequencing, DNA methylation, microRNA sequencing, and protein arrays were used to clarify the subtype and molecular mechanism of TNBC, and the datasets were deposited in public databases, such as The Cancer Genome Atlas (TCGA) that these data approved the heterogeneity of the clinical behavior [13]. Besides, these public datasets offer possibilities for surveying the molecular mechanism from different perspectives. Thus, profoundly understanding the molecular pattern of TNBC helps to conduct novel strategies to treat cancers.

In the current study, we identified differentially expressed genes (DEGs) by comparison between the genes expression profiles of samples from TNBC and non-TNBC patients. These genes were listed and underwent gene ontology (GO) and Kyoto Encyclopedia of Genes and Genomes pathway (KEGG) analysis. Protein-protein interaction (PPI) network and module screening and interrelation between pathways were performed. Next, the correlation between gene expression and survival was carried out that these data may shed light on further insight of TNBC and explored the potential targets for diagnosis, prognosis, and drug discovery.

## 2. Materials and Methods

### 2.1. Microarray Data

GSE76275 was composed of gene expression data using the Affymetrix U133 Plus 2.0 gene expression array from 265 samples, including 67 non-TNBC and 198 TNBC.

### 2.2. Identification of Differentially Expressed Genes (DEGs)

GEO2R, supplied by the National Center for Biotechnology Information, is an interactive web tool that was used to identify the DEGs between TNBC and non-TNBC samples. In the current study, genes with fold change ≥1.5 and p<0.01 were regarded as DEGs. Morpheus, a web-based tool, was used to draw the heatmap and the top 100 significantly changed genes (up- and downregulated genes) were presented.

### 2.3. Gene Ontology (GO) and Kyoto Encyclopedia of Genes and Genomes (KEGG) Pathway Enrichment Analysis of DEGs

Biological significance was explored by GO term enrichment analysis, biological process, cellular component, and molecular function included. Search Tool for the Retrieval of Interacting Genes/Proteins (STRING) version 10.5 was used by inputting the gene name of DEGs and exporting the results. P<0.05 was considered statistically significant. The Database for Annotation, Visualization and Integrated Discovery (DAVID) version 6.8 was used to infer the Kyoto Encyclopedia of Genes and Genomes (KEGG) pathways.

### 2.4. Protein-Protein Interaction (PPI) Network Building and Interrelation Analysis between Pathways

STRING database version 10.5 was applied to evaluate the protein-protein interaction (PPI) information. Network of DEGs was conducted using settings with experiments, textmining, database, coexpression, neighborhood, gene-fusion, and cooccurrence box checked. MCODE, a plug in Cytoscape version 3.5.0, was used to screen the modules from PPI network. Modules with MCODE score >3 and nodes number >3 were presented. Interrelation analysis between pathways was performed by ClueGo plug in Cytoscape version 3.5.0, using biological process terms/pathways and immune system process terms/pathways, and only those terms/pathways with p<0.05 were presented.

### 2.5. Survival Analysis

In this study, survival analysis refers to the Overall Survival Kaplan-Meier Estimate. Survival analysis was performed using datasets termed Breast Cancer (METABRIC, Nature 2012 & Nat Commun 2016) from cBioPortal database. The datasets used in this study were composed of 2509 breast cancers samples/patients, and, then, they were filtrated by the immunohistochemistry (IHC) status of estrogen receptor (ER), progesterone receptor (PR), and human epidermal growth factor receptor 2 (Her2). In total, 320 patients were selected that these patients suffered the so-called TNBC. Based on GO enrichment analysis and interrelation network built by ClueGO, gene names were submitted in cBioPortal, and survival analysis was carried out, of which those genes with Logrank Test p <0.05 were presented.

## 3. Results

### 3.1. Identification of Differentially Expressed Genes (DEGs)

**Figure 1:**
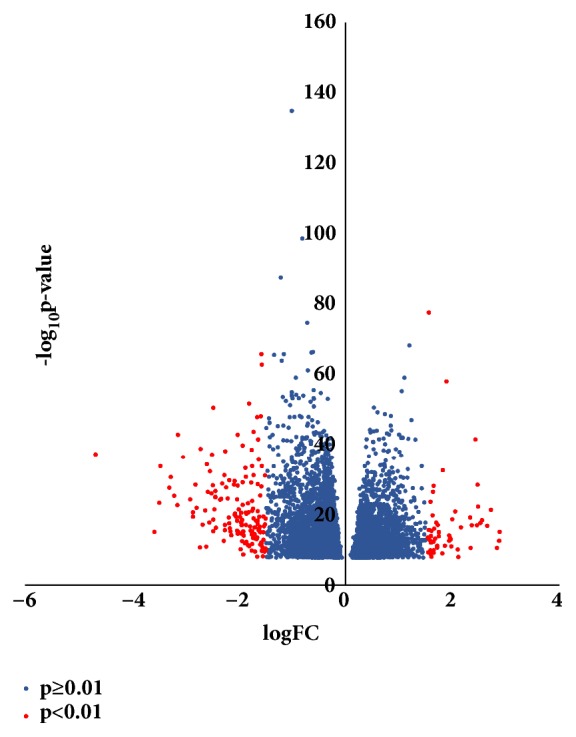
Volcano plot of 6891 genes. Red plots represented genes with fold change ≥1.5 or ≤-1.5, p<0.01. Blue plots represented the rest of the genes with no significant expression change.

**Figure 2:**
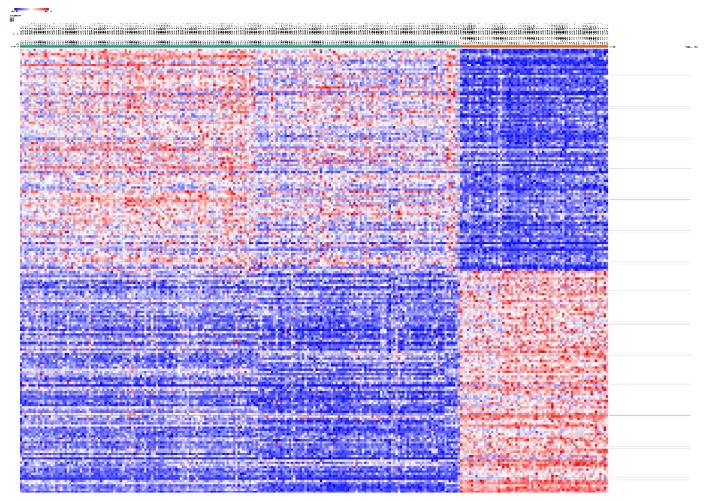
Heatmap of the top 100 differentially expressed genes (100 up- and 100 downregulated genes). Red ones represented upregulation and blue represented downregulation.

GSE76275 was selected and underwent differentially expressed genes (DEGs) analysis using GEO2R. 6891 genes were identified either up- or downregulated in all. Among them, 56 up- and 151 downregulated genes (207 in total) were designated and listed as significantly changed DEGs that expressed fold change ≥1.5 or ≤-1.5 and p<0.01. All 6891 genes were plotted that red ones represented 207 DEGs and blue ones were the rest of the genes, as shown in Figure 1. The expression levels of all the genes (6891) were demonstrated and top 100 genes were presented in the heatmap, and these genes were well clustered between non-TNBC and TNBC as shown in Figure 2.

### 3.2. Gene Ontology (GO) Term Enrichment Analysis and Kyoto Encyclopedia of Genes and Genomes (KEGG) Pathway Analysis of DEGs

All the significantly changed genes name was submitted to Search Tool for the Retrieval of Interacting Genes/Proteins (STRING). For the cellular component (CP), DEGs were significantly enriched in the extracellular region, extracellular exosome, vesicle, and membrane-bounded vesicle, as shown in Table 1. For the molecular function, DEGs were significantly enriched in RNA polymerase II regulatory region sequence-specific DNA binding, transcription regulatory region DNA binding, transcriptional activator activity, RNA polymerase II transcription regulatory region, transcription factor activity, RNA polymerase II core promoter proximal region sequence-specific binding, transcriptional activator activity, RNA polymerase II core promoter proximal region sequence-specific binding, transcription regulatory region sequence-specific DNA binding, RNA polymerase II transcription factor activity, sequence-specific DNA binding, core promoter proximal region sequence-specific DNA binding, and enhancer sequence-specific DNA binding, as shown in Table 2. For the biological process, DEGs were significantly enriched in neural crest cell development, neural crest cell differentiation, epithelial cell differentiation, growth, developmental growth, positive regulation of developmental process, epithelium development, regulation of cell proliferation, and epithelial cell development as shown in Table 3. All the DEGs were submitted to the Database for Annotation, Visualization and Integrated Discovery (DAVID), and the Kyoto Encyclopedia of Genes and Genomes (KEGG) analysis was performed and showed that genes are mainly involved in Oocyte meiosis, as shown in Table 4.

### 3.3. Construction of Protein-Protein Interaction Network and Analysis of Interrelation between Pathways

**Figure 3:**
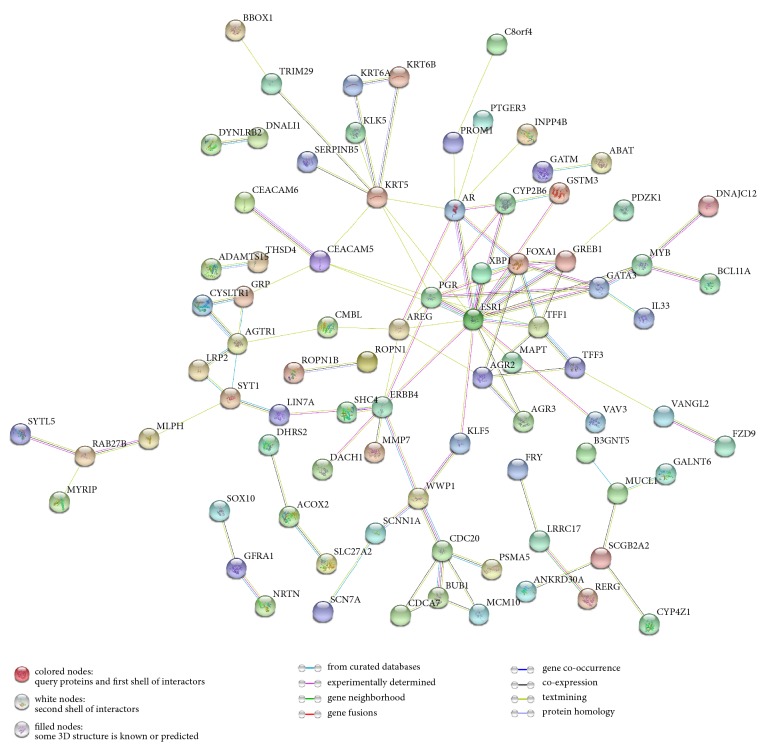
Construction of protein-protein interaction (PPI) network. PPI enrichment, p value=4.92e-14.

**Figure 4:**
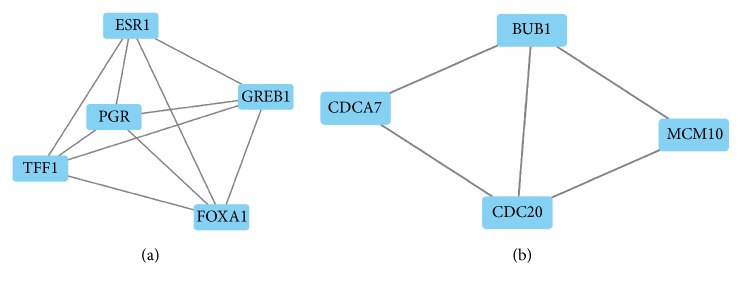
Modules inferred from protein-protein interaction network. (a) Module A; (b) module B. MCODE score >3, nodes >3.

**Figure 5:**
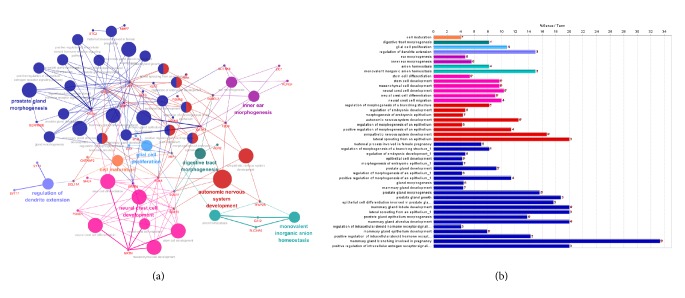
Interrelation analysis between pathways (biological process). (a) Interrelation between biological process pathways; (b) count numbers of genes involved in the identified pathways.

**Figure 6:**
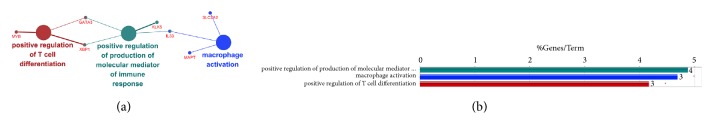
Interrelation analysis between pathways (immune system process). (a) Interrelation between immune system pathways; (b) count numbers of genes involved in the identified pathways.

All DEGs were submitted to STRING and network was presented. As shown in Figure 3, 168 nodes and 108 edges were identified, PPI enrichment p value=4.92e-14. Based on the PPI network, modules were identified. As shown in Figures 4(a), 4(b), and 2 modules were inferred that estrogen receptor 1 (ESR1), progesterone receptor (PGR), growth regulation by estrogen in Breast Cancer 1 (GREB1), trefoil factor 1 (TFF1), and forkhead box A1 (FOXA1) formed module A while cell division cycle associated 7 (CDCA7), BUB1 mitotic checkpoint serine/threonine kinase (BUB1), minichromosome maintenance 10 replication initiation factor (MCM10), and cell division cycle 20 (CDC20) formed module B. Interrelation analysis was conducted by accessing the biological process and immune system process in ClueGO. All the DEGs were mainly enriched in prostate gland morphogenesis, inner ear morphogenesis, cell maturation, digestive tract morphogenesis, autonomic nervous system development, monovalent inorganic anion homeostasis, neural crest cell development, regulation of dendrite extension and glial cell proliferation, immune system process termed T cell differentiation, regulation of immune response, and macrophage activation. Most of the genes were involved in two or more processes as shown in Figures 5 and 6.

### 3.4. Survival Analysis

**Figure 7:**
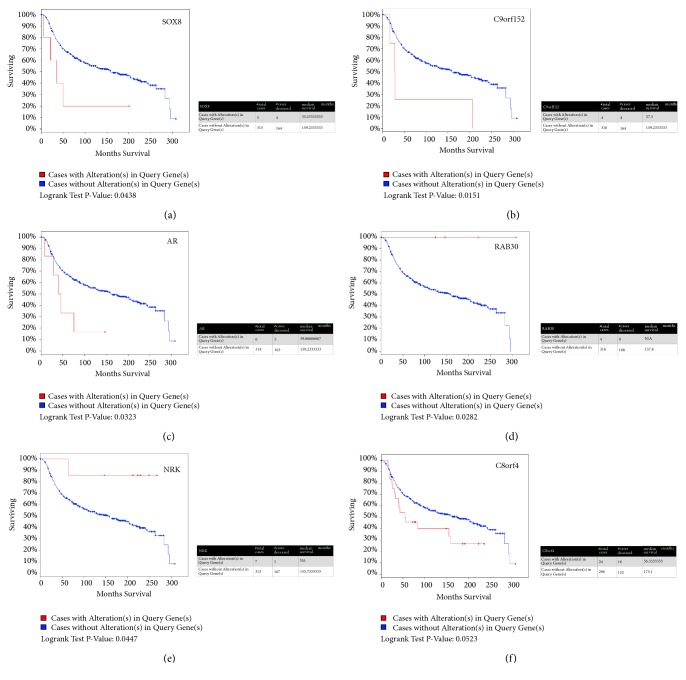
Overall Survival Kaplan-Meier Estimate of all DEGs in 320 TNBC from TCGA datasets termed Breast Cancer (METABRIC, Nature 2012 & Nat Commun 2016). Red line represents cases with alterations. Blue line represents cases without. (a) SOX8, p=0.0438; (b) C9orf152, p=0.0151; (c) AR, p=0.0323; (d) RAB30, p=0.0282; (e) NRK, p=0.0447; (f) C8orf4, p=0.0523.

**Figure 8:**
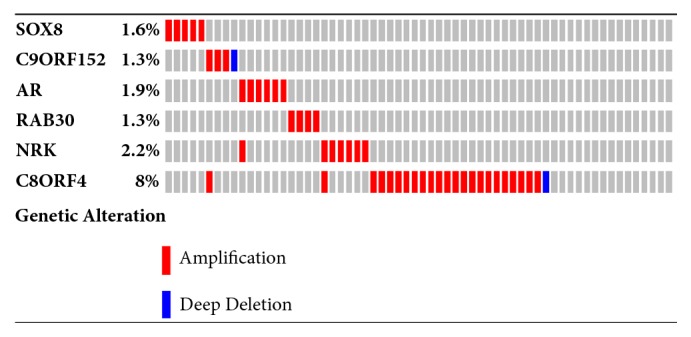
Oncoprint of genes. Every column represents a sample/patient. Red represents amplification; blue represents deep deletion.

All the DEGs underwent survival analysis using cBioPortal datasets termed Breast Cancer (METABRIC, Nature 2012 & Nat Commun 2016). 320 TNBCs were selected from 2509 breast cancer samples/patients by filtrating the immunohistochemistry (IHC) status of ER/PR/Her2. Among all the DEGs, SRY-Box 8 (SOX8), androgen receptor (AR), and Chromosome 9 Open Reading Frame 152 (C9orf152) significantly shortened the life expectancy (p<0.05) (Figures 6(a), 6(b), and 6(c)) while Nik Related Kinase (NRK) and RAS oncogene family 30 (RAB30) extended it (p<0.05) (Figures 6(d) and 6(e)). Besides, Chromosome 8 Open Reading Frame 4 (C8orf4) with p=0.0523, to some extent, shortens it (Figure 7(f)). Oncoprint showed that 47 altered in 320 sequenced cases/patients as shown in Figure 8.

## 4. Discussion

In this study, DEGs between samples/patients of TNBC and non-TNBC were identified and GO, PPI network, interrelation between pathways, and survival analysis were conducted. In total, 56 upregulated and 151 downregulated genes with p<0.01 and fold change≥1.5 or ≤-1.5 were listed. Based on these DEGs, bioinformatics analysis was conducted.

Firstly, cell component enrichment (GO) analysis showed that these DEGs mainly located in the extracellular region, exosome, and vesicle (Table 1). The term “extracellular vesicles” (EV) comprises several types of vesicles that are involved in drug resistance, increased proliferation, invasiveness, and cancer-induced immunosuppression [14]. Cancer-derived EVs have gained increasing attention as biomarkers and therapeutic targets with molecular cargo compared to single-molecule biomarker on circulating tumors. An elevated number of EVs have been found in the peripheral blood of samples/patients [15–17] and these EVs (type and numbers) could be used as diagnostic tool. Some of these genes identified in the present study have been documented with cancer progression but the mechanisms still need to be clarified. Within these genes (gene list not shown), for instance, 4-aminobutyrate aminotransferase (ABAT), involved in the cell component termed extracellular region (GO.0005576), low mRNA expression led to an accumulation of beta-alanine and shortened relapse-free survival [18].

Secondly, molecular function enrichment (GO) analysis showed that DEGs mainly promoted the transcription factor activity and RNA polymerase II core promoter proximal region sequence-specific binding (Table 2). Phosphorylation of RNA polymerase II large subunit is required for initiation and elongation of transcription; however, inhibition of this process leads to cell death in preclinical models of TNBC [19]. Besides, the molecular classification of TNBC includes the luminal androgen receptor (AR) subtype and this receptor was also identified (gene list not shown). In fact, approximately 10-15% of TNBCs express androgen receptor (AR) [20, 21]. The molecular function of AR in the progress of TNBC remains unclear while promising data targeting inhibition of TNBC is triggering much more interests [22–24]. Another identified gene, for example, X-Box Binding Protein 1 (XBP1), forms a complex with hypoxia-inducible factor 1-alpha (HIF1*α*) that recruits RNA polymerase II to HIF1*α* target genes [25].

Thirdly, biological process (GO) enrichment analysis showed that DEGs were mainly involved in neural crest cell and epithelial cell differentiation and development (Table 3). The epithelial–mesenchymal transition (EMT) refers to the process by which the epithelial cells lose polarity and cell-cell adhesion and obtained migratory and invasive properties to become mesenchymal stem cells that are multipotent stromal cells that can differentiate into a variety of cell types. For migration to begin, neural crest cells must undergo a process called delamination that involves a full or partial EMT [26]. In fact, the SOX family, especially SOX10, identified in the present study, regulates cancer stem cell properties of TNBC cells [27] and participate in early determination and migration [28–30].

Above all, these DEGs played an important role in a variety of biological processes and some of these genes were well documented but the mechanisms related to TNBC still need to be clarified.

Next, the network of DEGs and the interrelation of pathways were analyzed in the current study. Mostly, genes interacted directly or indirectly with others. Those genes termed “node” and the line connected called “edge” were drawn based on literature mining, experimental evidence, and databases (as shown in Figure 3). Module screening showed that, within the network, core genes might form a subnetwork that plays an important role in the development of TNBC. As shown in Figures 3 and 4(a), there were 12 experimentally determined edges connected with estrogen receptor 1 (ESR1). Although TNBC with low expression of ESR1 (fold change=-3.49 compared to non-TNBC in our data), it still played a pivotal role in TNBC. In fact, ESR1-methylation represented higher probability in TNBC than non-TNBC [31] and variants in different loci showed a correlation with high risk of suffering diseases [31]. Trefoil factor 1 (TFF1) expression (fold change=-2.68 in our data) exhibited inverse association with tumor size and histological grade [32]. Growth regulation by estrogen in Breast Cancer 1 (GREB1) (fold change=-2.81 in our data) interacts with estrogen receptor (ER) in half of ER positive primary breast cancers [33]; however, how could it interact with ESR1 in TNBC remains unknown. Coexpression of Forkhead box A1 (FOXA1) with androgen receptor (AR) could be used as a biomarker for the identification of subtypes of TNBC [34] and promotes tumor cell proliferation [35]. Recent studies have shown that loss of cell division cycle associated 7 (CDCA7) leads to the inhibition of EMT and stemness in TNBC cells [36] while it expressed highly in TNBC (fold change=1.64 in our data). Cell division cycle 20 (CDC20) (fold change=1.65 in our data) and securin were reported to be promising candidates in the treatment of TNBC [37] that they could promote cell migration and invasion [38]. Loss of BUB1 mitotic checkpoint serine/threonine kinase (BUB1) could reduce cancer stem cell potential of breast cancer cell line [39]. Minichromosome maintenance 10 replication initiation factors (MCM10) were recognized as biomarker for identification of subtype TNBC [40]. Above all, these genes either promote or inhibit the progression of TNBC that the complex interaction between genes need to be explored. Next, the interrelation analysis was conducted. Within these pathways (as shown in Figures 5 and 6), immune system process analysis showed that T cell differentiation, regulation of immune response, and macrophage activation interacted with each other through the key genes such as GATA Binding Protein 3 (GATA3) (regulates tumor microenvironment [41]), XBP1 (promotes triple-negative breast cancer by regulating the HIF1*α* pathway [42]), interleukin-33 (IL-33) (promotes breast cancer growth and progression [43]), MYB protooncogene, transcription factor (MYB), kallikrein related peptidase 5 (KLK5), microtubule associated protein tau (MAPT), and solute carrier family 7 member 2 (SLC7A2). These results demonstrated that the node genes might be a key player in the immune system in the progression of TNBC.

Finally, in order to figure out whether these DEGs related to the life expectation, all the DEGs were submitted to cBioPortal and conducted survival analysis using the datasets termed METABRIC, Nature 2012, and Nat Commun 2016. Amplification of SRY-Box 8 (SOX8) significantly shortens the survival of patients, as shown in Figure 7(a). Up to now, there was no report that clarified the exact role of SOX8 in TNBC. However, the member of the SOX family, SRY-Box 9 (SOX9), was reported to play a critical role in the TNBC cell proliferation, migration, and invasion [44]. SRY-Box 10 (SOX10) induced nestin expression regulates cancer stem cell properties of TNBC cells [27]. Besides, SOX10 preferentially expressed in TNBC that it could be used as a promising diagnostic marker [45, 46]. SOX8 was also expressed in TNBC and used as a signature of the subtype of TNBC [4]. However, the mechanism of SOX8 contribution for TNBC initiation and progression remains unknown. Chromosome 9 Open Reading Frame 152 (C9orf152) is highly expressed in TNBC but without any studies indicating the relevance with TNBC that knock-down of this gene could help to figure out the function in TNBC. Androgen receptor (AR), as has been well documented, was another highly expressed gene that showed significant correlation with life expectation of patients [47, 48]. RAB30, member RAS oncogene family (RAB30), and Nik Related Kinase (NRK) were identified and could be of benefit for the patients as shown in Figures 7(c) and 7(d), but how these genes exert effect in TNBC needs further investigation, of which, nevertheless, Logrank Test p value was 0.0523, not statistically significant, and Chromosome 8 Open Reading Frame 4 (C8orf4) represented tendency of decreasing the survival. The topic of what the exact relation between the high expression of C8orf4 and survival is needs further exploration.

In conclusion, comprehensive bioinformatics analysis of gene expression profiles of TNBC compared with non-TNBC was conducted; thus, the key genes and pathways were identified. All the DEGs might participate in a variety of pathways in the initiation, progression, and invasion of TNBC. The study provides a set of targets for further research of molecular mechanisms and biomarkers. Other than bioinformatic exploration, corroborative bench work was needed to confirm the function of genes identified.

## Figures and Tables

**Table 1:** Cellular component (GO) enrichment analysis result of differentially expressed genes (DEGs) with fold change ≥1.5 or ≤-1.5 and p<0.01.

**#pathway ID**	**pathway description**	**observed gene count**	**false discovery rate**
**GO.0005576**	extracellular region	59	0.00607

**GO.0044421**	extracellular region part	50	0.00967

**GO.0070062**	extracellular exosome	41	0.00967

**GO.0031982**	vesicle	46	0.0402

**Table 2:** Molecular function (GO) enrichment analysis result of differentially expressed genes (DEGs) with fold change ≥1.5 or ≤-1.5 and p<0.01.

**#pathway ID**	**pathway description**	**observed gene count**	**false discovery rate**
**GO.0000977**	RNA polymerase II regulatory region sequence-specific DNA binding	16	0.001

**GO.0044212**	transcription regulatory region DNA binding	19	0.001

**GO.0001228**	transcriptional activator activity, RNA polymerase II transcription regulatory region sequence-specific binding	12	0.00502

**GO.0000982**	transcription factor activity, RNA polymerase II core promoter proximal region sequence-specific binding	12	0.00599

**GO.0001077**	transcriptional activator activity, RNA polymerase II core promoter proximal region sequence-specific binding	10	0.00714

**GO.0000976**	transcription regulatory region sequence-specific DNA binding	15	0.00808

**GO.0000981**	RNA polymerase II transcription factor activity, sequence-specific DNA binding	14	0.0459

**GO.0000987**	core promoter proximal region sequence-specific DNA binding	10	0.0459

**GO.0001158**	enhancer sequence-specific DNA binding	5	0.0459

**Table 3:** Biological process (GO) enrichment analysis result of differentially expressed genes (DEGs) with fold change ≥1.5 or ≤-1.5 and p<0.01.

**#pathway ID**	**pathway description**	**observed gene count**	**false discovery rate**
**GO.0014032**	neural crest cell development	6	0.0203

**GO.0014033**	neural crest cell differentiation	6	0.0203

**GO.0030855**	epithelial cell differentiation	15	0.0203

**GO.0040007**	growth	14	0.0203

**GO.0048589**	developmental growth	12	0.0203

**GO.0051094**	positive regulation of developmental process	23	0.0203

**GO.0060429**	epithelium development	22	0.0203

**GO.0042127**	regulation of cell proliferation	27	0.0239

**GO.0002064**	epithelial cell development	9	0.0382

**Table 4:** KEGG pathway analysis

**Category**	**KEGG_PATHWAY**
**Term**	hsa04114: Oocyte meiosis

**Count**	4

%	0.016343875

**P Value**	0.0711977

**Genes**	PGR, AR, BUB1, CDC20

**List Total**	62

**Pop Hits**	109

**Pop Total**	6910

**Fold Enrichment**	4.089967446

**Bonferroni**	0.999835961

## Data Availability

Data Availability For GEO data downloading, readers can visit the website https://www.ncbi.nlm.nih.gov/ and choose “GEO datasets” in the drop-down list.For gene enrichment analysis, a web based tool DAVID (https://david.ncifcrf.gov/) was used.For inferring the protein-protein interaction, we use web based tool named STRING (https://string-db.org/) according to the instruction. For drawing the network, Cytoscape 3.5 was used. For life span expectation analysis, we visit cBioportal.

## Supplementary Materials

Supplementary Materials A “step-by-step” introduction for analyzing the GSE76275 was offered for readers to follow.
