# Supplementary material for: Identification of Key Genes and Pathways in Triple-Negative Breast Cancer by Integrated Bioinformatics Analysis
Source: Biomed Res Int. 2018 Aug 2;2018:2760918. doi: 10.1155/2018/2760918 (PMC6098886; doi:10.1155/2018/2760918)
Supplement: Supplementary Materials — A “step-by-step” introduction for analyzing the GSE76275 was offered for readers to follow. [file 2760918.f1.docx]

**A step by step induction for analyze GSE76275**

1. **Identification of differentially expressed genes (DEGs)**

**
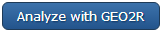
Step 1:** Visit the website <https://www.ncbi.nlm.nih.gov/geo/query/acc.cgi?acc=GSE76275>. Click the button

**Step 2:** Click the drop-down list named “Define groups” to define groups of the non-TNBC and the TNBC by selecting samples according to the description offered by the submitter of GSE76275.

**Step 3:** When all samples were defined, click the link “save all results”, and then, a list of genes, p values, fold change and other related results would come out. Save all to a txt file and reopen it with excel.

**Step 4:** Copy the column of gene name, p value and fold change to a new sheet. Filter and select genes both p<0.01 and fold change≥1.5.

Thus, we get the so-called differentially expressed genes (DEGs) list in hand.

1. **Gene ontology (GO) Kyoto Encyclopedia of Genes and Genomes (KEGG) pathway enrichment analysis of DEGs**

**Step 1:** visit the website <https://string-db.org/> and change the model to “multiple proteins”. Paste all the DEGs names to the box and select organism to *Homo sapiens* and click search.

*Notes:* In the next page, one should go to the end of the page and click “continue”, otherwise you won’t get the full results.

**Step 2:** click “Analysis” and the **Gene ontology (GO) list composed of Biological Process (GO), Molecular Function (GO) and Cellular Component (GO) was printed**. Download the tables in the box of “Save/Export”.

**Setp 3:** Visit the website <https://david.ncifcrf.gov/> and click “Start Analysis”. First, paste the gene list of the DEGs. Second, click the drop-down list of “Select Identifier” and select “OFFICIAL_GENE_SYMBOL”. Third, designate the list type by choose “Gene List” and then “Submit List”. Click “Functional Annotation Chart”, and click “Pathways”, remain the box of “KEGG_PATHWAY” checked, and click “chart” just like shown in the screenshot, finally, **a table of “KEGG_PATHWAY” was presented**.
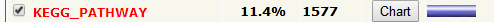


1. **Protein-protein interaction (PPI) network building and interrelation analysis between pathways**

**STEP 1:** visit the website <https://string-db.org/> and change the model to “multiple proteins”. Paste all the DEGs names to the box and select organism to Homo sapiens and click search with default settings. **A picture of PPI network was showed** and one can export it.

*Notes:* In the next page, one should go to the end of the page and click “continue”, otherwise you won’t get the full results. Settings that one could s

**STEP 2:** export the current network by clicking
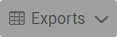
 in the upper panel, and you will see
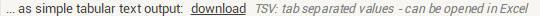


Click the link “download”, you will get a txt file named “string_interactions.tsv”. Open Cytoscape 3.5.1 (there might be updated version) and import the network by clicking the icon
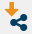
 in the control bar of the software and selecting the “string_interactions.tsv”. Open the app plug in the software by clicking “Apps” in the top panel of the control bar and select MCODE. Click
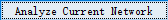
 botton, and modules analysis results come out in the right window of the software. One could edit and export the figures in Cytoscape. **This the so-called modules analysis.**

**STEP 3:** open another app plug called ClueGO by clicking “Apps” in Cytoscape and select ClueGO or install the plug by downloading it from the inside link. Generally, these plugs are free and you just need to offer an email with *edu* suffix when you register in it. Copy and paste the DEGs list into the box of “Load Marker List(s)”. Then, choose which “Ontologies/Pathways” you would like to evaluate. In this manuscript, we choose “GO-Biological Process” and “GO-Immune System Process” respectively in the box of ClueGO Settings with “show only Pathways with pV≤” checked. Default setting was pV≤0.05. Click “Start” botton, all pathways will be presented. Suspend mouse pointer on the icons in the control panel on the right and find “Show all genes from all pathways/terms” and click. Thus, **interrelation analysis of pathways were carried out.**

1. **Survival analysis**


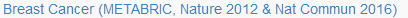
**STEP 1:** visit the website <http://www.cbioportal.org/data_sets.jsp> and click “Data Sets” in the top of the page. Select data sets.

Click “Add chart” drop down list. Select “ER Status”, “HER2 Status”, “PR Status” respectively. Then, at the bottom of the page there are three charts added. Click the corresponding “negative” part of the three charts respectively. Thus, **320** **TNBC were selected among these 2509 breast cancer patients.**

**STEP 2:** find the table of DEGs and copy genes one by one into the box of query genes. For example, you can input SOX8 in the box, and “query”. In the new page, you will find “Survival” tab control and click it, **a survival analysis result comes out.** Overall Survival Kaplan-Meier Estimate was done and you can see the Logrank Test p-value is 0.0438. In the manuscript, only those Logrank Test p-value <0.05 was presented.
